# Establishment of Serum Cholesterol Sulfate Reference Intervals and Evaluation of Its Diagnostic and Metabolic Associations in Chinese Adults

**DOI:** 10.3390/diagnostics16182953

**Published:** 2026-09-12

**Authors:** Yin Liu, Xucong Ji, Xiaoqian Yu, Hongmei Zhang, Xinhua Dai, Zhiguang Su

**Affiliations:** 1 Department of Laboratory Medicine, West China Hospital, Sichuan University, Chengdu 610041, China; 2 Sichuan Clinical Research Center for Laboratory Medicine, and Clinical Laboratory Medicine Research Center of West China Hospital, Chengdu 610041, China; 3 Department of Pain Management, West China Hospital, Sichuan University, Chengdu 610041, China; 4 Institute of High-Altitude Medicine, Center for High Altitude Medicine, High Altitude Medicine Key Laboratory of Sichuan Province, West China Hospital, Sichuan University, Chengdu 610041, China; 5 State Key Laboratory of Biotherapy, Frontiers Science Center for Disease-Related Molecular Network, West China Hospital, Sichuan University, Chengdu 610041, China

**Keywords:** cholesterol sulfate, reference interval, Alzheimer’s disease, Crohn’s disease, metabolic biomarkers, LC-MS/MS

## Abstract

**Background/Objectives:** Cholesterol sulfate (CS) is a multifunctional signaling molecule implicated in diverse physiological and pathological processes. Its clinical translation as a biomarker is hindered by the lack of established reference intervals, unclear disease-specific alterations, and undefined relationships with routine laboratory parameters. This study aimed to establish a serum CS reference range for Chinese populations, evaluate its associations with various diseases and clinical laboratory parameters, and define its clinical diagnostic utility. **Methods:** A total of 642 subjects were enrolled, comprising 372 healthy controls and 270 patients with one of six diseases: Alzheimer’s disease, osteoporosis, obesity, type 2 diabetes mellitus (T2DM), non-alcoholic fatty liver disease (NAFLD), or Crohn’s disease. Serum CS concentrations were quantified using LC-MS/MS. Reference intervals were established using non-parametric methods. Univariate analyses included Spearman’s rank correlation, Mann–Whitney U test, and receiver operating characteristic (ROC) curve analysis; multivariate analysis employed a gamma-family generalized linear model (GLM) with a log-link function, incorporating age, sex, clinical laboratory parameters, and disease status as covariates. The age-by-sex interaction was examined to assess synergistic effects. Incremental diagnostic value of CS was evaluated using the DeLong test and likelihood ratio test. **Results:** The serum CS reference interval for healthy individuals was 0.55–2.24 mg/L (males: 0.60–2.25 mg/L; females: 0.54–2.20 mg/L), with a significant sex difference. The age-by-sex interaction effect was significant (two-way ANOVA interaction *p* = 0.005; GLM interaction β = 0.102, *p* = 0.002). Stratified analysis revealed a significant positive correlation between age and CS in males, but no such association in females. In univariate analysis, CS correlated significantly with total cholesterol (TC), non-HDL-C, hemoglobin A1c (HbA1c), and other biomarkers; CS levels were elevated in Alzheimer’s disease and reduced in Crohn’s disease. In the multivariate GLM, only TC and male sex emerged as independent significant determinants of CS, whereas HbA1c and NAFLD showed borderline effects; no disease group retained independent significance. After adjustment for TC and sex, the residual area under the curves (AUCs) of CS for all diseases were close to 0.50, and DeLong’s test indicated no incremental diagnostic value beyond the baseline model (all *p* > 0.70). **Conclusions:** This study is the first to establish a serum CS reference range for the Chinese population. Serum CS levels are independently regulated by TC and sex, with a significant age-by-sex interaction. The associations between CS and disease status were largely mediated by confounding from lipid profiles and age. CS is not suitable as an independent disease biomarker but may serve as a supplementary indicator for assessing lipid metabolism—particularly in male populations, where it may indirectly reflect age-related changes in cholesterol metabolism.

## 1. Introduction

Cholesterol sulfate (CS) is a major sterol sulfate in the human circulation and ranks among the most abundant steroid sulfates in the body [1,2]. It is synthesized primarily by hydroxysteroid sulfotransferase 2B1b (SULT2B1b), which catalyzes the sulfation of the 3β-hydroxyl group of cholesterol. CS is widely distributed across cell membranes, the stratum corneum, platelets, and the central nervous system, where it functions as a multifunctional signaling molecule involved in diverse physiological and pathological processes. In the skin, CS plays a critical role in keratinocyte differentiation, maintenance of epidermal barrier integrity, and normal desquamation [3,4]. Within the immune system, CS exerts dual modulatory effects: it regulates T-cell activity by inhibiting DOCK-2 and promoting T-cell receptor (TCR) dimerization [5], while simultaneously impairing TCR signaling through disruption of T-cell microvilli, thereby alleviating T-cell-mediated hypersensitivity responses [6]. In the nervous system, CS exerts neuroprotective effects by modulating energy metabolism, activating Akt signaling, and suppressing oxidative stress [7]. In addition, CS serves as a negative-feedback regulator of cholesterol homeostasis, influencing both cholesterol biosynthesis and efflux [8].

Given its broad biological activities, CS has been implicated in the pathogenesis of various diseases. In neurodegeneration, CS accelerates amyloid-β (Aβ) plaque formation [9] and promotes tau hyperphosphorylation, contributing to neurofibrillary tangle pathology [10,11,12,13]. In the gastrointestinal tract, intestinal SULT2B1 expression is markedly upregulated in patients with ulcerative colitis, and CS facilitates colonic epithelial repair via the CS–NPC2–SREBP2–cholesterol synthesis axis [14]. CS also acts as a negative-feedback signal for hepatic gluconeogenesis and lipogenesis [15]. Furthermore, CS promotes pancreatic β-cell proliferation and protects against stress-induced apoptosis, thereby preserving functional β-cell mass and mitigating diabetes progression [16].

Despite these pathophysiological implications, the clinical translation of CS as a biomarker faces three major bottlenecks. First, normal reference intervals for serum CS have not been established, and the effects of sex and age on its distribution remain insufficiently characterized, making it difficult to define abnormal CS values and clinically relevant thresholds in clinical practice. Second, the direction and magnitude of CS alterations across different diseases remain unclear, and whether these changes are influenced by age and sex is unknown. This may introduce bias or lead to erroneous interpretations of disease-related effects [17]. Third, the relationship between CS and routine clinical laboratory parameters, including glycemic, lipid, hepatic, and renal indices, remains poorly defined, particularly after adjustment for age and sex. This leaves the metabolic implications and clinical significance of CS largely unresolved.

In this study, we aimed to systematically characterize the clinical utility and metabolic relevance of CS through a progressive analytical framework. Specifically, we sought to establish normative reference intervals for serum CS, to determine the direction and magnitude of CS alterations across six representative diseases (Alzheimer’s disease, osteoporosis, type 2 diabetes mellitus, non-alcoholic fatty liver disease, obesity, and Crohn’s disease), to elucidate the relationships between CS and routine clinical laboratory parameters, and to evaluate the incremental diagnostic value of CS beyond conventional risk factors. Through this comprehensive approach, we expect to provide a rigorous evidence base for the clinical interpretation and potential application of serum CS.

## 2. Material and Methods

### 2.1. Study Population

The study protocol was approved by the Ethics Committee of West China Hospital (approval no. 2024062). This retrospective study enrolled a total of 642 participants, including healthy individuals undergoing routine health check-ups and patients with one of six target diseases who were either hospitalized or seen at outpatient clinics at West China Hospital between 2025 and 2026 (Supplementary Table S1). The healthy control group comprised 372 subjects, all of whom were confirmed to be free of cardiovascular, metabolic, autoimmune, or malignant diseases through comprehensive physical examinations and thorough review of available routine laboratory data (including fasting blood glucose, serum creatinine, estimated glomerular filtration rate, liver enzymes, and lipid profiles) to exclude common asymptomatic conditions such as prediabetes, early chronic kidney disease, and subclinical hepatic dysfunction. The disease groups included osteoporosis (*n* = 71), Alzheimer’s disease (*n* = 40), obesity (*n* = 40), type 2 diabetes mellitus (T2D, *n* = 40), non-alcoholic fatty liver disease (NAFLD, *n* = 40), and Crohn’s disease (*n* = 39). All patients met the corresponding international diagnostic criteria for their respective conditions. The six diseases were selected to represent distinct pathophysiological categories with established or plausible links to CS metabolism, including neurodegeneration [9], intestinal epithelial repair [14], hepatic gluconeogenesis and lipid metabolism [15], and β-cell function [16]. Exclusion criteria were deliberately designed to eliminate conditions that could independently and substantially alter CS levels, thereby avoiding confounding of the disease-specific comparisons. Accordingly, we excluded patients with X-linked ichthyosis (a disorder of steroid sulfatase deficiency causing markedly elevated CS), severe hepatic failure (given the liver’s primary role in CS synthesis), major coexisting illnesses that could confound the analysis (e.g., patients with both obesity and T2DM, or T2DM with overt diabetic nephropathy), and pregnancy (due to profound hormonal effects on steroid metabolism).

### 2.2. Reagents, CS Standards, and Sample Preparation

LC-MS-grade methanol was purchased from Knowles (Itasca, IL, USA). Ultrapure water was obtained from a Milli-Ro 60 Water System (Millipore, Milford, MA, USA). Bovine serum albumin (BSA) was sourced from Shanghai Aladdin Biochemical Technology Co., Ltd. (Shanghai, China). CS (purity ≥ 98%) and deuterated CS (CS-D7, internal standard, purity ≥ 99%) were purchased from Shanghai Bide Pharmaceutical Co., Ltd. (Shanghai, China).

Stock solutions of CS and CS-D7 were prepared in methanol at the concentration of 1 mg/L. These stock solutions were serially diluted with methanol to generate eight working solutions for calibration curves, validation, and sample analyses. All stock solutions were stored at −20 °C until use.

Aliquots (30 µL) of standard solutions, quality control samples, and serum specimens were transferred into 1.5 mL tubes. Subsequently, 10 µL of the internal standard working solution (0.5 mg/L) and 110 µL of methanol were added to each tube. The mixtures were vortex-mixed for 5 min and centrifuged at 15,000 rpm for 10 min at 25 °C. The resulting supernatants were collected and injected into the LC-MS/MS system for analysis.

### 2.3. Chromatographic and Mass Spectrometric Conditions

Liquid chromatography–tandem mass spectrometry (LC-MS/MS) analyses were performed on a high-performance LC-MS/MS system (Thermo Fisher Scientific, Waltham, MA, USA) operated in multiple reaction monitoring (MRM) mode. The mass spectrometer was equipped with an electrospray ionization (ESI) source operated in negative-ion mode.

Chromatographic separation was achieved on a Robusta C18 column (100 × 2.1 mm, 3 μm; SepaChrom, Rho, Italy), maintained at 35 °C with a flow rate of 0.2 mL/min. The mobile phase comprised 10 mM ammonium formate in water (solution A) and methanol (solution B). A gradient elution was performed as follows: 75% to 95% B over 12 min, held for 6 min, returned to 75% B in 1 min, followed by 6-min re-equilibration. For both the analyte and the internal standard, one quantifier and one qualifier transition were monitored.

The mass spectrometric parameters were set as follows: nebulizer gas (GAS1) and auxiliary gas (GAS2) at 60 psi each, curtain gas at 10 psi, source temperature at 600 °C, ion spray voltage at −4500 V, and declustering potential at −100 V. The Q1 scan range was m/z 97–465.1, and the product ion scan (MS2) was performed with collision energies of 20–40 eV over a range of m/z 50–500.

### 2.4. Validation of CS Measurement

The method was validated in terms of linearity range, extraction efficiency, accuracy, precision, and matrix effect in accordance with the principles of the ICH M10 guideline on bioanalytical method validation [18].

Linearity was evaluated over the concentration range of 0.05–10 mg/L, using eight calibration points (0.05, 0.1, 0.2, 0.5, 1, 2, 5, and 10 mg/L). The internal standard (IS) concentration was 0.5 mg/L. Calibrators were prepared by spiking standard solutions into methanol, and the calibration procedure was repeated across five independent analytical batches. Calibration curves were constructed by plotting the peak area ratio of CS to the IS against nominal concentrations. Linear regression analysis with a 1/x^2^ weighting factor was applied to minimize the influence of higher-concentration standards and ensure maximum accuracy at the lower concentrations of the calibration curve, where most samples are expected. The quality of the calibration curves was assessed by examining the determination coefficient (r^2^) and the mean accuracy of calibrators both within and across analytical batches.

Extraction efficiency was assessed by comparing the peak area of CS and the internal standard (IS) spiked into the matrix before extraction (A) and with those spiked into the matrix after extraction (B). At least five replicates of quality control (QC) samples were analyzed at three concentration levels: low QC (LQC, 0.1 mg/L), middle QC (MQC, 0.5 mg/L) and high QC (HQC, 2 mg/mL). The mean extraction efficiency at each QC level was required to have a coefficient of variation (CV) ≤ 20%.

Matrix effect, defined as the influence of co-eluting substances from the biological matrix on the analyte signal, was evaluated by comparing the peak areas of CS and IS spiked into extracted blank matrix (post-extraction spike, B) with those of neat standard solutions at equivalent concentrations (C). The matrix factor (MF) for each compound was calculated as the ratio B/C. An MF < 1 indicates ion suppression, whereas MF > 1 indicates ion enhancement. To account for variability in the IS, the normalized matrix factor was derived as the ratio of the analyte MF to the IS MF. This assessment was performed at the same three QC concentration levels, with at least five replicates each. The coefficient of variation (CV) of the normalized matrix factors was required to be ≤15%, in accordance with bioanalytical validation guidelines.

### 2.5. Statistical Analysis

Statistical analyses were conducted using SPSS software (version 23.0, IBM Corp., Armonk, NY, USA), with supplementary analyses conducted in Python 3.13 using the SciPy, statsmodels, and scikit-learn libraries. Continuous variables are expressed as mean ± standard deviation (SD) or median with interquartile range (IQR), as appropriate. Categorical variables are presented as frequencies (percentages). Normality was assessed using the Shapiro–Wilk test. All tests were two-sided, and a *p*-value < 0.05 was considered statistically significant unless otherwise specified.

#### 2.5.1. Reference Interval Establishment

Given the slight skewness in CS concentration distribution, reference intervals were estimated using the nonparametric 2.5th–97.5th percentile method, with 90% confidence intervals (CIs) calculated via bootstrap resampling (2000 iterations) [19]. The necessity of stratification by sex was evaluated using the Mann–Whitney *U* test, while age-group partitioning was assessed using the Kruskal–Wallis test.

#### 2.5.2. Age–Sex Interaction Analysis

Two complementary approaches were adopted: (1) Two-way ANOVA, with sex and age groups (<30, 30–39, 40–49,5 0–59, ≥60 years) as fixed factors, to test the significance of the interaction term; and (2) a gamma Generalized Linear Model (GLM) with a log-link function, incorporating standardized age, gender, and their interaction term as predictors to estimate the magnitude and direction of the interaction effect. Subsequently, stratified analysis was performed to calculate Spearman’s correlation coefficient between age and CS separately for males and females.

#### 2.5.3. Correlation Analysis

Spearman’s rank correlation coefficient (ρ) was used to assess the crude correlations between CS and various clinical laboratory indicators. For partial correlation analysis, the effects of age and sex were first removed via ordinary least squares (OLS) regression, and Spearman’s ρ was then computed on the residuals.

#### 2.5.4. Multivariate Regression Modeling

A gamma-family GLM with a log-link function was employed, with CS concentration as the dependent variable and the following independent variables: age, sex, total cholesterol (TC), high-density lipoprotein cholesterol (HDL-C), triglycerides (TGs), creatinine (CREA), alanine aminotransferase (ALT), direct bilirubin (DBIL), total bilirubin (TBIL), hemoglobin A1c (HbA1c), gamma-glutamyl transferase (GGT), albumin (ALB), glucose (GLU), uric acid (UA), and six disease groups (with healthy controls as the reference group). All continuous variables were standardized (via z-transformation); effect sizes are expressed as exp(β), representing the multiplicative change in CS per one-standard-deviation increase in the predictor. Multicollinearity was assessed using the variance inflation factor (VIF), with a threshold of VIF < 5 considered acceptable. As a sensitivity analysis, the same model was refitted using a Gaussian family with a log-link.

#### 2.5.5. Diagnostic Performance Evaluation

Receiver operating characteristic (ROC) curves were constructed for six distinct diseases under three models: (1) CS alone, (2) a baseline model (TC + gender), and (3) a full model (TC + sex + CS). The DeLong test was used to compare area under the curve (AUC) differences between the baseline model and full models, while the likelihood ratio test assessed the incremental predictive value of CS within the full model. Additionally, the AUC the residual CS value (after adjusting for TC and sex) was calculated.

#### 2.5.6. Matched Subgroup Analysis

Patients with Alzheimer’s disease (aged 50–70 years) and Crohn’s disease (aged 25–45 years) were each matched 1:1 with healthy controls of the corresponding age range using the Kallor matching method (with a caliper of ±5 years of age and exact match on sex). CS concentration and CS/TC ratios between cases and matched controls were compared using the paired Wilcoxon signed-rank test.

## 3. Results

### 3.1. Basic Characteristics of the Study Population

**Figure 1:**
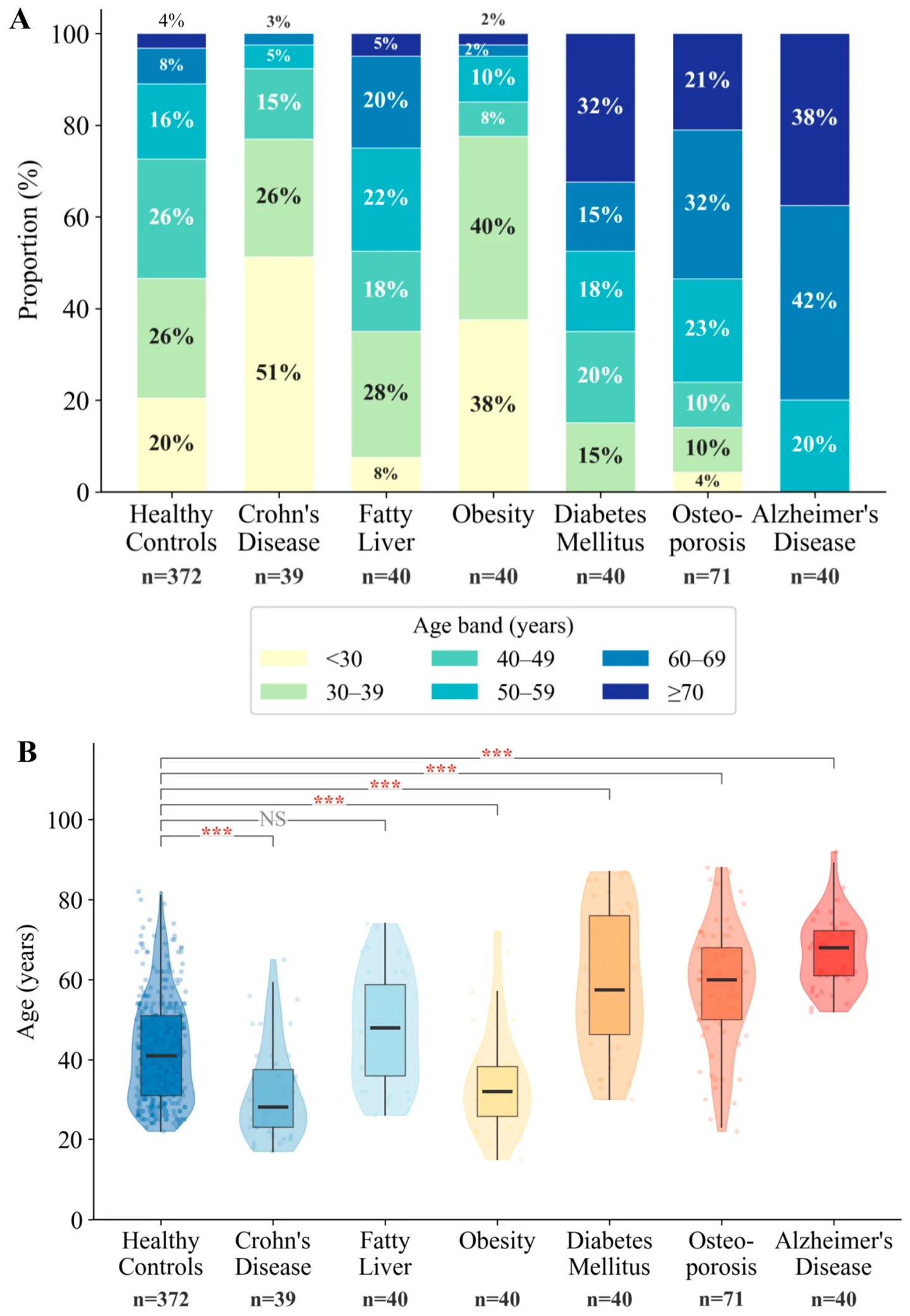
Study population age distribution. (**A**) Stacked bar chart (100%) showing the proportional distribution of six age bands (<30, 30–39, 40–49, 50–59, 60–69, ≥70 years) across healthy controls and six disease groups. (**B**) Violin plots with overlaid boxplots (IQR, median) and jittered individual data points; sample sizes are given below each group label. Pairwise comparisons of age between each disease group and HC were performed using two-sided Mann–Whitney U tests with Bonferroni correction for six simultaneous tests. *** *p* < 0.001, NS, not significant. IQR, interquartile range.

A total of 642 participants were enrolled, comprising 372 healthy controls (181 males, 191 females) and 270 patients across six disease groups. Baseline demographic and biochemical characteristics are summarized in Supplementary Table S1. Age showed the widest intergroup variation (median age range: 28–68 years) (Figure 1A). Compared with healthy controls, age was significantly higher in the osteoporosis, Alzheimer’s disease, obesity, T2DM, and Crohn’s disease groups (all *p* < 0.001), but not in the NAFLD group (*p* = 0.071) (Figure 1B). Sex distribution remained balanced across all groups. Lipid profiles differed significantly among groups (TC, TG, HDL-C, LDL-C, and non-HDL-C; all *p* < 0.001). The T2DM group exhibited a characteristic diabetic dyslipidemia pattern (elevated TG and reduced HDL-C), whereas the Crohn’s disease group had the lowest TC level (3.55 mmol/L). Glycemic indices (GLU and HbA1c) also showed intergroup differences (*p* < 0.001), attributable primarily to the T2DM group (GLU 7.34 mmol/L, HbA1c 7.00%). Liver function markers (ALT, AST, GGT, TBIL) and renal function markers (CREA, eGFR, UA, UREA) all showed significant intergroup variation (*p* values ranging from <0.001 to 0.021). Specifically, the NAFLD and obesity groups had substantially elevated transaminases; the T2DM and Alzheimer’s disease groups exhibited higher creatinine and lower eGFR; and the Crohn’s disease group—being the youngest age—presented the highest eGFR. Overall, the six disease groups demonstrated marked clinical heterogeneity across nearly all measured dimensions. Given this heterogeneous baseline profile, any observed associations between CS and disease status or clinical indices must be interpreted with careful adjustment for potential confounders.

### 3.2. Method Validation for CS Quantification

As shown in Table 1, five calibration curves were constructed across different analytical runs (B1-B5). The back-calculated concentrations of calibration standards were within ±15% of nominal values at all levels, and the precision (CV%) was below 15% for all points. The slope values of the five calibration curves ranged from 0.11 to 1.05, with a mean of 1.06 (SD = 0.10), indicating good consistency in analytical response across runs. The coefficient of determination (R^2^) was ≥ 0.998 for all curves (mean R^2^ = 0.998, SD = 0.001), demonstrating excellent linearity across the calibration range. These results confirm the accuracy and precision of the calibration procedure.

We next evaluated the extraction efficiency and matrix effect. As shown in Table 2, the absolute extraction recovery of CS was 107.8% (CV% = 7.8%) at the low QC level (0.1 mg/L), 98.48% (CV% = 1.3%) at the medium QC level (0.5 mg/L), and 111.17% (CV% = 1.1%) at the high QC level (2 mg/L), indicating consistent and satisfactory recovery across all QC levels. The slightly higher CV at the low concentration (7.80%) compared with the medium (1.33%) and high (1.09%) levels is attributable to more pronounced matrix effects, adsorption loss during sample preparation, and greater response variability at the lower end of the calibration range. Nevertheless, this value remained well below the acceptance limit of ±15% (or ±20% at the LLOQ) specified in the ICH M10 guideline [18], confirming that the method retains adequate precision and reliability even at the lowest concentration tested.

Regarding the matrix effect, the relative biases for serum matrices 1, 2, and 3 were −0.08%, −1.88%, and 0.44%, respectively (Table 3). These minimal deviations indicate negligible relative matrix effects, further supporting the robustness of the method for application to clinical serum samples.

### 3.3. Serum CS Concentrations in Healthy Individuals

**Figure 2:**
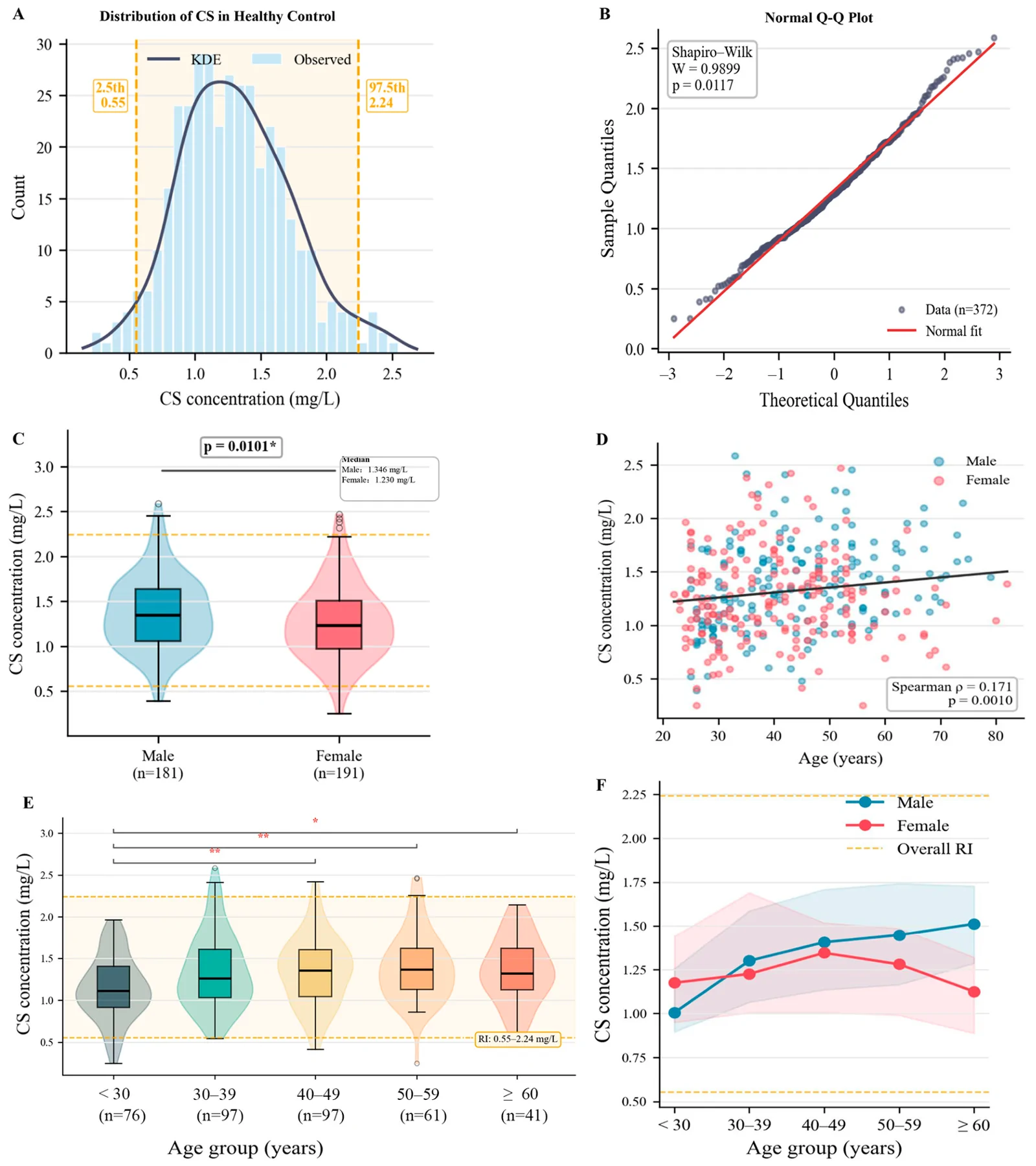
Distribution of serum CS concentrations and establishment of reference intervals in 372 healthy controls. (**A**) Histogram overlaid with kernel density curve showing the overall distribution. (**B**) Q–Q plot against the theoretical normal distribution. (**C**) Boxplots stratified by age group, with Bonferroni-corrected significance annotations, * *p* < 0.05. (**D**) Scatter plot with regression line illustrating the relationship between CS and age. (**E**) Boxplot/violin plot comparing CS levels between males and females, * *p* < 0.05, ** *p* < 0.01. (**F**) Median trend lines (with IQR shaded) for males and females across age groups. Dashed lines (C, E and F) indicate the 2.5th (lower) and 97.5th (upper) percentile reference limits. Q–Q, quantile–quantile; IQR, interquartile range.

A total of 372 healthy volunteers (approximate 1:1 male-to-female ratio) were enrolled in this study. The overall mean serum CS concentration was 1.32 ± 0.42 mg/L, with a median of 1.29 mg/L (IQR: 1.02~1.59 mg/L) (Supplementary Table S1). The frequency distribution of CS concentrations (Figure 2A) deviated slightly from normality according to the Shapiro–Wilk test (W = 0.990, *p* = 0.012, skewness = 0.35) (Figure 2B). Given this mild skewness and the potential influence of demographic factors observed in preliminary screening, we first examined the independent effects of sex and age on CS levels, followed by an assessment of their interaction, to provide a comprehensive statistical basis for establishing population-specific reference intervals.

Sex-stratified analysis revealed that the median CS concentration in males (1.35 mg/L, IQR: 1.06–1.64) was approximately 8.4% higher than that in females (1.23 mg/L, IQR: 0.97–1.51) (Figure 2C), confirming a significant sex-related difference in CS levels.

Next, we analyzed the association between age and CS levels. Serum CS concentration showed a weak positive correlation with age (Figure 2D). When subjects were stratified into five age groups (<30, 30–39, 40–49, 50–59, and ≥60 years), CS levels increased by approximately 0.06 mg/L per decade, with significant intergroup differences detected by the Kruskal–Wallis test (*p* = 0.003) (Figure 2E). Post-hoc comparisons showed that the <30 years group had significantly lower CS levels than the 40–49, 50–59, and ≥60 years groups, whereas no significant differences were observed among the groups aged ≥30 years (Figure 2E). Given the effect size and sample size constraints for establishing robust age-stratified reference intervals per CLSI EP28-A3c recommendations [20], we did not derive age-specific intervals. Instead, the <30 years group—which exhibited significantly lower CS levels than all older groups—should be evaluated using the lower end of the reference range, and clinicians should be aware that age-related increases in CS are predominantly confined to the male population.

To formally evaluate whether the effect of age on CS levels differs by sex, two complementary analytical approaches were employed (Supplementary Table S2). (1) Two-way ANOVA, with sex and age as fixed factors, revealed a significant main effect of sex (F = 4.43, *p* = 0.036), a highly significant main effect of age (F = 6.58, *p* < 0.001), and a significant sex-age interaction (F = 3.77, *p* = 0.005), indicating that the age-related trajectory of CS differs between males and females. (2) A gamma GLM with a log-link function, incorporating standardized age (Age-z), sex, and their interaction as predictors, yielded a significant interaction coefficient (β = 0.102, (exp(β) = 1.107, *p* = 0.002), suggesting that for each standard deviation increase in age, the relative increase in CS concentration among males is approximately 10.7% higher than that among females. Stratified analyses further corroborated this interaction: a significant positive correlation between age and CS was observed in males, but not in females (Supplementary Table S3 and Figure 2F). Collectively, these findings indicated that the age-related increase in CS is predominantly driven by the male subgroup.

Based on the significant sex difference and the age-by-sex interaction, sex-specific reference intervals were established. The overall 2.5th–97.5th percentile reference interval was 0.55–2.24 mg/L, with sex-specific intervals of 0.60–2.25 mg/L for males and 0.54–2.20 mg/L for females (Supplementary Table S4).

### 3.4. Univariate Analysis of Serum CS Levels with Clinical Parameters and Disease Status

**Figure 3:**
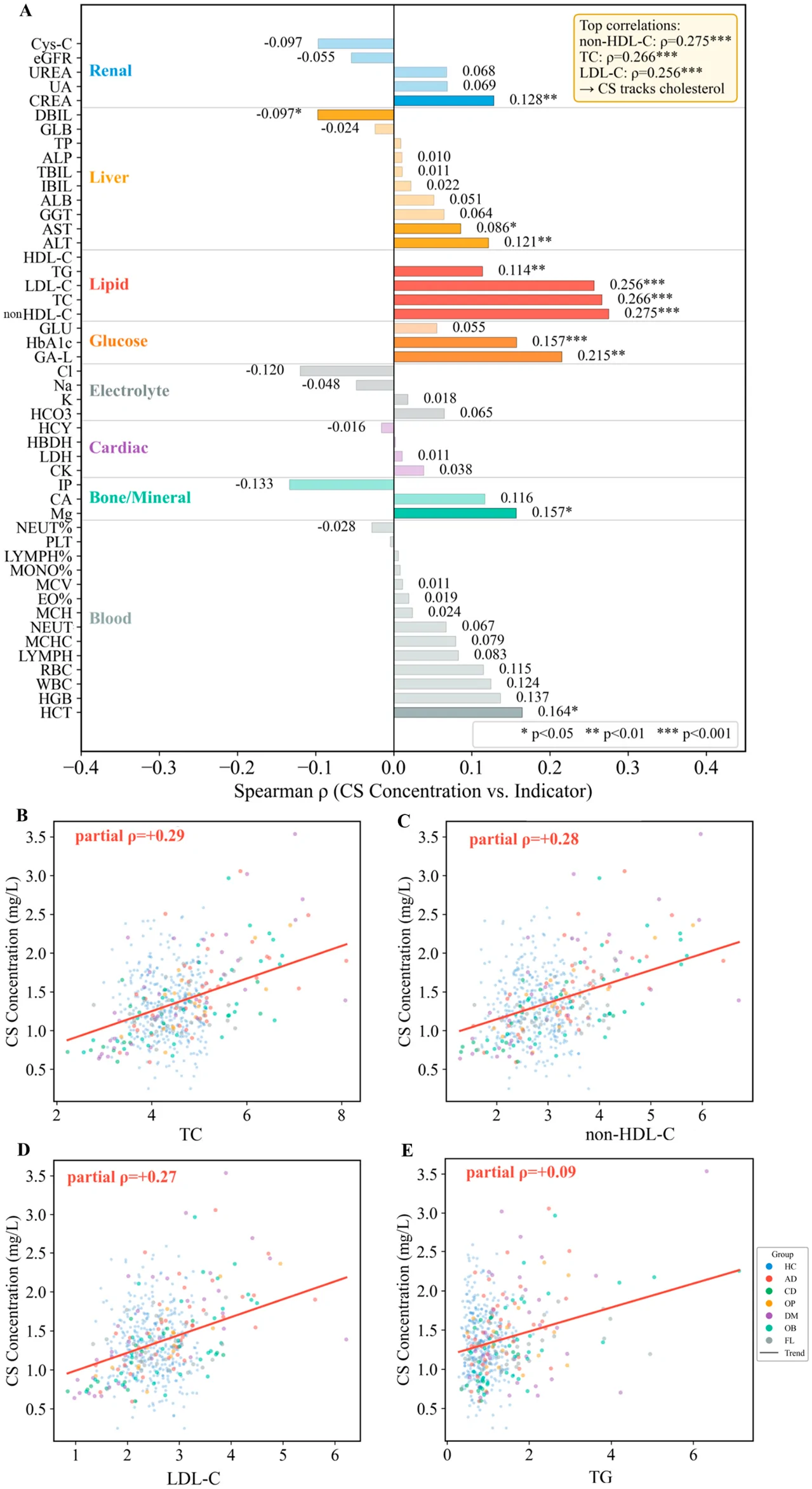

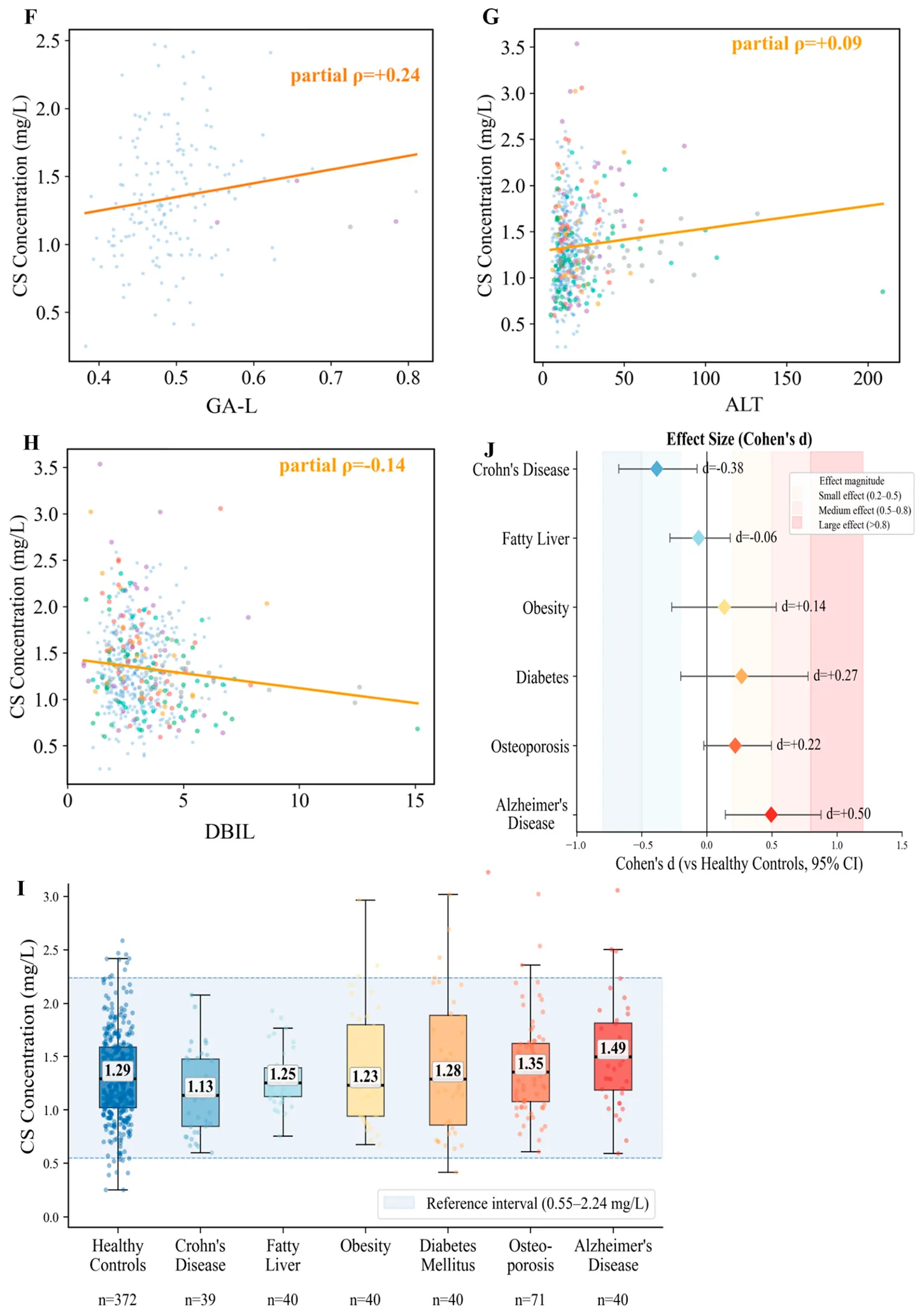
Correlation analysis between serum CS concentration and clinical laboratory parameters. (**A**) Heatmap of Spearman correlation coefficients between serum CS and various laboratory indices; (**B**–**H**) age+sex-adjusted partial Spearman ρ correlation analyses of CS with Top 7 significantly associated clinical parameters; (**I**) boxplots with scatter overlay showing the distribution of serum CS concentrations across diseases; (**J**) CS effect size (Cohen’s d) for each disease group.

A Spearman correlation analysis was performed between serum CS levels and 48 clinical laboratory parameters across eight major categories (Figure 3A). Twelve parameters showed significant correlations with CS (*p* < 0.05) (Figure 3A, Supplementary Table S5). Lipid-related indices (TC, LDL-C, non-HDL-C, and TG) exhibited the strongest associations (correlation coefficients ρ = 0.11–0.28). Significant correlations were also observed for renal function (CREA), liver function (ALT, AST and DBIL), and glycemic markers (HbA1c and glycated albumin, GA-L).

To determine whether these associations were confounded by age and sex, we performed ordinary least squares (OLS) regression of CS and each parameter against these two demographic variables, followed by Spearman partial correlation analysis on the residuals. After adjustment, seven parameters remained independently and significantly correlated with CS (*p* < 0.05). Three lipid parameters (non-HDL-C, TC, LDL-C, and TG) exhibited slightly strengthened associations, while hepatic function-associated parameter ALT retained significant positive correlations (Figure 3B–G and Supplementary Table S5). Notably, DBIL, which had shown only a weak negative correlation before adjustment, demonstrated a markedly stronger negative association afterwards, suggesting that age and sex had previously masked its true relationship (Figure 3H and Supplementary Table S5). In contrast, the apparent correlations of HbA1c, HCT, AST, Mg, and CREA with CS were no longer significant after adjustment (Supplementary Table S5), confirming that their crude associations were predominantly driven by age/sex confounding. Collectively, these findings indicate that serum CS exhibits robust, independent positive correlations with cholesterol-related indices (non-HDL-C, TC, and LDL-C) and the glycemic markers (GA-L), whereas its associations with CREA, HCT, ALT, and other parameters are largely attributable to differences in age structure.

We next compared CS concentrations across the six disease groups. The median CS levels, in descending order, were: Alzheimer’s disease (1.49 mg/L) > osteoporosis (1.35 mg/L) > healthy controls (1.29 mg/L) > diabetes mellitus (1.28 mg/L) > NAFLD (1.25 mg/L) > obesity (1.23 mg/L) > Crohn’s disease (1.13 mg/L) (Figure 3I), with overall intergroup differences reaching statistical significance (Supplementary Table S1). Without adjustment for potential confounders, the patients with Alzheimer’s disease had the highest median CS concentration (1.494 mg/L), corresponding to Cohen’s d of +0.495 relative to controls (*p* = 0.012). Conversely, the patients with Crohn’s disease had the lowest median CS concentration (1.134 mg/L), with d of –0.384 (*p* = 0.019) (Figure 3J and Supplementary Table S6).

### 3.5. Multivariate Analysis and Validation of Serum CS-Associated Factors

To distinguish true independent effects from spurious associations confounded by demographic or clinical variables in the univariate analysis, a gamma-family GLM with a log-link function was constructed. The model includes age, sex, TC, TG, HDL-C, HbA1c, GLU, CREA, ALT, GGT, ALB, TBIL, UA, and six disease groups (with healthy controls as the reference group). The model was fitted using complete data from 434 subjects (pseudo-R^2^ = 0.153, AIC = 473.9, dispersion = 0.098); all variables had variance inflation factor (VIF) < 3, indicating no significant multicollinearity. As a sensitivity analysis, the same model was refitted using a Gaussian distribution, yielding highly consistent results (18 of the 19 variables retained the same direction of significance).

**Figure 4:**
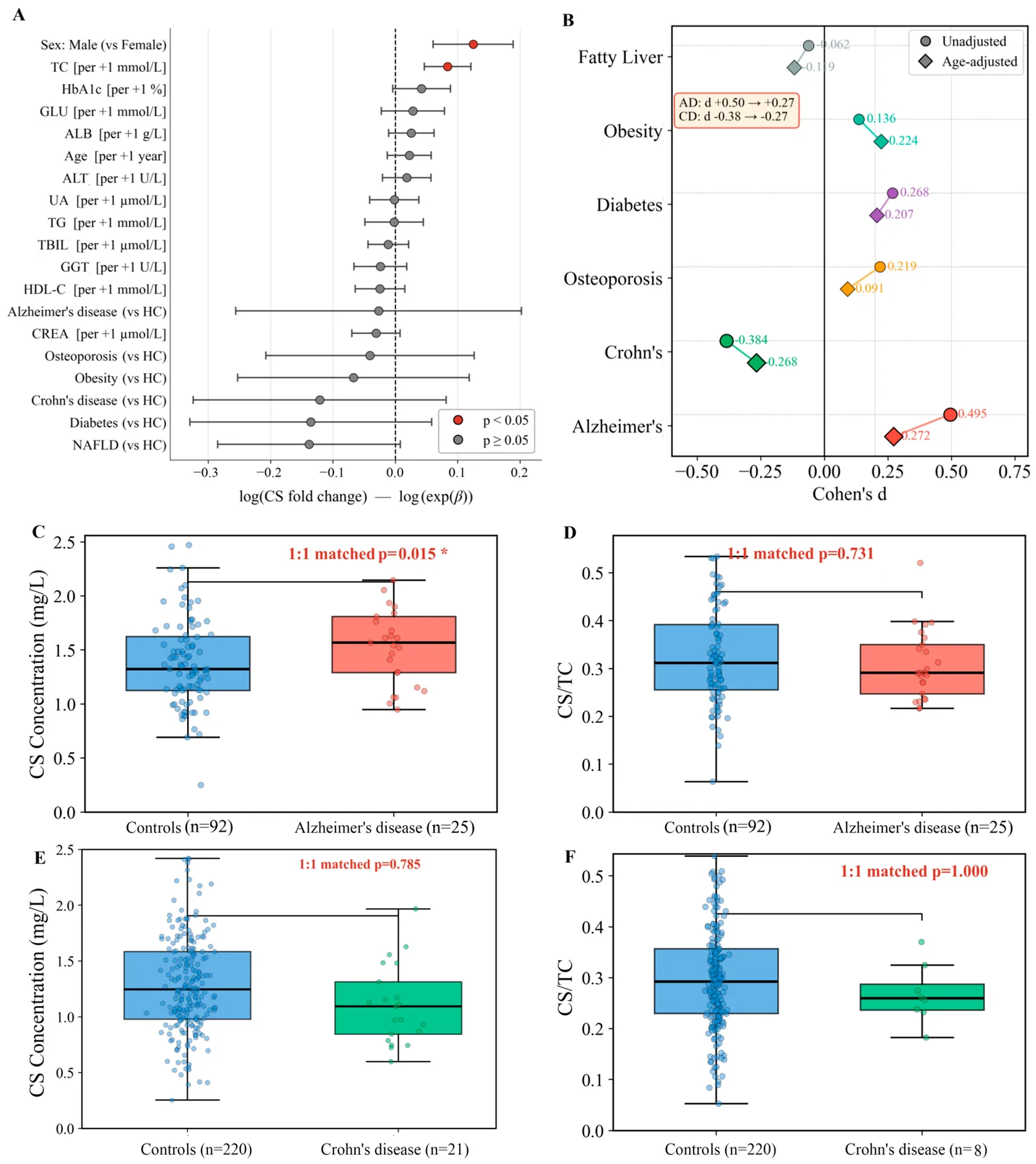
Multifactorial analysis validation of serum CS. (**A**) Forest plot showing the effect sizes of each GLM variable on CS concentration (Gamma distribution, log-link function), Gamma family, log link), *n* = 434, pseudo-R^2^ = 0.153. Dashed line = no effect (fold change = 1.0). Bars = 95% CIs. Continuous predictors are reported per clinical unit (see brackets); (**B**) comparison of effect sizes (Cohen’s d) for CS concentrations before and after age adjustment; (**C**–**F**) comparison of CS concentration and CS/TC ratio between age-matched subgroups; (**C**,**D**) Alzheimer’s disease patients versus matched healthy controls, * *p* < 0.05; (**E**,**F**) Crohn’s disease patients versus matched healthy controls.

The GLM results identified only TC and male sex as independent significant predictors of CS concentration (Supplementary Table S7). For TC, each 1-SD increase corresponded to a 1.088-fold increase in CS (*p* < 0.0001); in original units, each 1 mmol/L increase in TC corresponded to a 1.134-fold increase (*p* < 0.0001), representing the strongest positive modulator. For male sex, the CS ratio for males versus females was 1.133 (*p* = 0.0001), indicating approximately 13.3% higher CS levels in males, independent of all covariates. All other variables were not statistically significant in the multivariate model, with HbA1c (×1.043, *p* = 0.075) and NAFLD (×0.871, *p* = 0.064) showing only borderline effect. The independent effects of all six disease groups were non-significant (*p* = 0.172–0.819), indicating that the elevated CS in Alzheimer’s disease and reduced CS in Crohn’s disease observed in univariate analysis were largely attenuated after adjusting for confounding factors such as TC, age, and sex (Figure 4A).

Next, we systematically compared how the effect of each variable changed across analytical levels (Supplementary Table S8). Three distinct patterns emerged: (1) True independent effect: both TC and male sex remained significant across all three levels, confirming their roles as independent determinants of CS. (2) Confounded pseudo-correlation: biomarkers such as HbA1c, CREA, HCT, Mg, and AST showed significant crude correlations that became non-significant after adjustment for age/sex and remained so in the fully adjusted model, indicating they were largely driven by age-related confounding. (3) Disease-associated false associations: Alzheimer’s disease (univariate d = +0.495, *p* = 0.012) and Crohn’s disease (d = –0.384, *p* = 0.019) were significant in univariate comparisons but substantially attenuated in the GLM (Alzheimer’s disease: ×0.974, *p* = 0.819; Crohn’s disease: ×0.886, *p* = 0.242), suggesting that these disease-related differences are primarily mediated by TC and age (Figure 4B and Supplementary Table S8).

To further validate these findings, matched subgroup analyses were performed. For Alzheimer’s disease patients (aged 50–70 years), 25 pairs were matched 1:1 with healthy controls of the same age and sex (age difference: 0.20 ± 0.49 years). After matching, the CS concentration remained significantly higher in patients with Alzheimer’s disease than in controls (paired Wilcoxon *p* = 0.015), whereas the CS/TC ratio showed no difference (*p* = 0.731). This pattern suggests that the elevated CS level in Alzheimer’s disease may reflect a residual disease effect that is abolished after normalization by TC (Figure 4C,D). For Crohn’s disease patients (aged 25–45 years), 21 pairs were matched (age difference: 0.00 years); neither the CS concentration (*p* = 0.785) nor the CS/TC ratio (only 8 valid pairs available, limiting statistical power) showed a significant difference, confirming that the reduced CS level in Crohn’s disease was primarily driven by age-related confounding (Figure 4E,F).

### 3.6. Clinical Utility of CS

**Figure 5:**
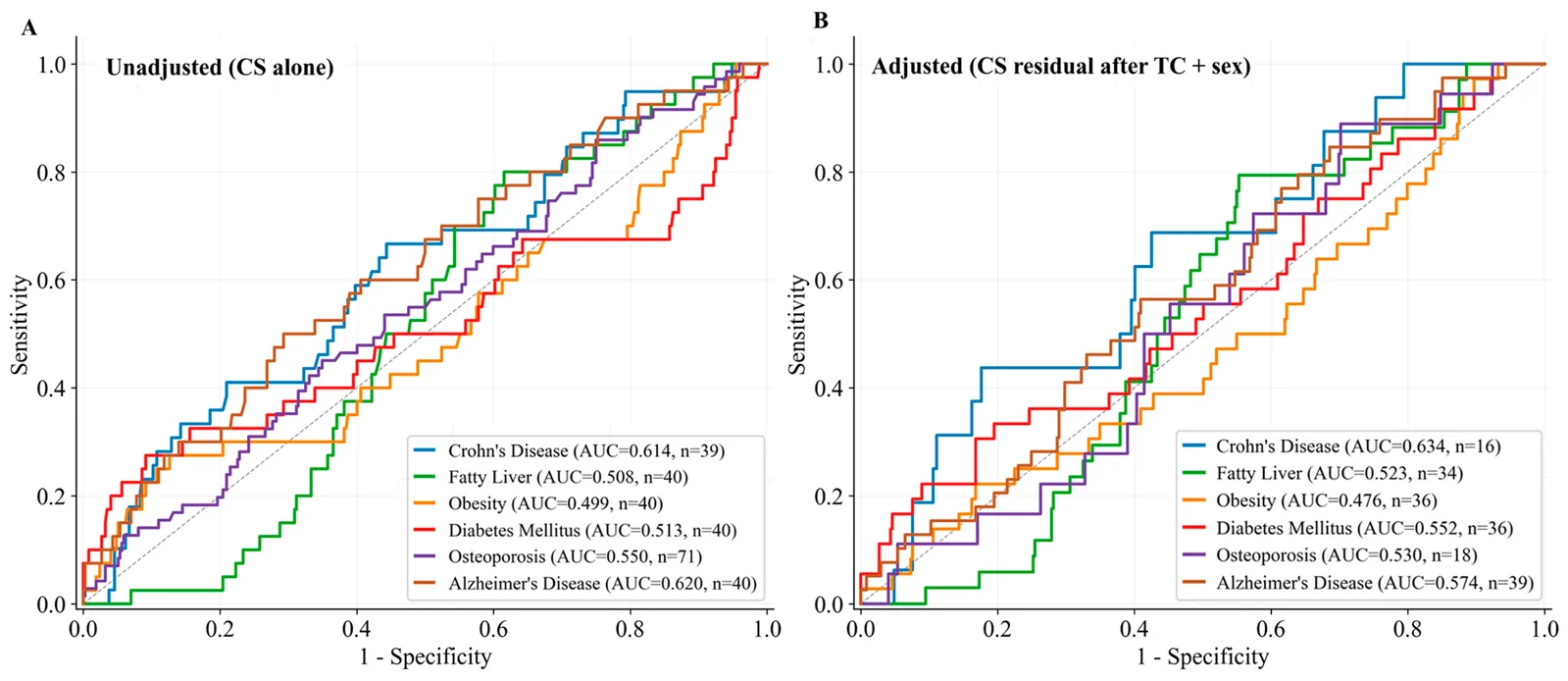
Diagnostic performance of serum CS for six diseases versus healthy controls before and after adjustment for TC and sex. (**A**) ROC curves using unadjusted CS alone; (**B**) ROC curves using the residual of CS after linear regression on TC and sex. Each ROC curve compares one disease group to healthy controls; legends report AUC, disease group size, and healthy control group size. CS, cholesterol sulfate; TC, total cholesterol; ROC, receiver operating characteristic.

We first evaluated the diagnostic performance of serum CS alone (i.e., the CS-only model) for six diseases using unadjusted ROC analysis. CS achieved its highest AUC values for Alzheimer’s disease (0.621) and Crohn’s disease (0.614); however, both remained below the clinically acceptable threshold of 0.70. For all other diseases, AUC values were close to 0.50 (range: 0.499–0.550), indicating negligible discriminative utility (Figure 5A). Building on the multivariate findings—which indicated that the intergroup differences observed in univariate analyses were largely mediated by confounders such as TC and age—we constructed two additional ROC models for each disease: a baseline model (TC + sex) and a full model (TC + sex + CS). The DeLong test was used to compare AUCs between the two models, and the residual AUC of CS after accounting for TC and sex was also calculated. The results showed that the ΔAUC between the full and baseline models was minimal across all diseases (−0.005 to +0.031), with all DeLong test *p*-values > 0.70, indicating that CS provided no additional diagnostic value beyond the baseline model. The residual AUC of CS consistently remained near 0.50 (range: 0.476–0.634); specifically, it shifted from 0.620 to 0.574 for Alzheimer’s disease and from 0.614 to 0.634 for Crohn’s disease, showing no consistent improvement in discriminative ability. The likelihood ratio test further supported this conclusion (all *p* > 0.05 (Figure 5B and Supplementary Table S9).

## 4. Discussion

Serum CS concentrations in humans have been reported to vary widely across studies, with ranges spanning from 1.75 to 350 mg/L, largely due to differences in analytical methods, sample sizes, and population characteristics [21,22,23,24,25,26,27]. This study established, for the first time, serum CS reference intervals for a Chinese population, with an overall range of 0.55–2.24 mg/L and clear evidence supporting sex-specific partitioning (males: 0.60–2.25; females: 0.54–2.20 mg/L), in line with earlier reports [26,27]. This sex disparity may reflect androgen-mediated regulation of SULT2B1b expression or higher baseline cholesterol metabolism in men, although the underlying mechanisms remain to be elucidated. The reference intervals established here strictly followed the nonparametric method recommended by the CLSI EP28-A3c guideline [20], providing a robust baseline for clinical application. Compared with sporadically reported CS ranges in the literature [27], our study benefits from a larger sample size (*n* = 372) and employs bootstrap resampling to compute 90% confidence intervals, ensuring greater precision. Nevertheless, these reference intervals are derived from cross-sectional data and have not yet been validated in an independent cohort; their external generalizability warrants further investigation.

Serum CS showed a weak positive correlation with age, with an approximate increase of 0.06 mg/L per decade. However, this age effect exhibited striking sex-specific heterogeneity. In males, CS correlated strongly positively with age, with median values rising from 1.005 mg/L in those <30 years to 1.513 mg/L in those ≥60 years. In contrast, females showed no significant age correlation and displayed a non-monotonic pattern, peaking in the 40–49 age group (median 1.345 mg/L) and declining thereafter. This trajectory coincides with the average onset of menopause (around age 50), suggesting that estrogen-dependent modulation of SULT2B1b/cholesterol homeostasis attenuates after menopause [2], thereby curtailing or reversing the age-related rise in CS. These findings carry important clinical implications: age must be carefully considered when interpreting CS levels in males, whereas menopausal status may be more relevant than chronological age in females. Further age-stratified analyses showed that the <30-year group had significantly lower CS than all older age groups, with no differences among groups aged ≥30 years, pointing to age 30 as a potential threshold for CS stabilization.

Alzheimer’s disease is pathologically characterized by Aβ plaque deposition and hyperphosphorylation of tau protein [28]. In our cohort, CS demonstrated a medium effect on Alzheimer’s disease in unadjusted analyses, which was attenuated after adjustment for age. Age-matched subgroup analysis (50–70 years) further revealed a residual elevation of CS in Alzheimer’s disease patients compared with matched controls. However, this effect was no longer significant when CS was expressed as a ratio to TC (CS/TC) (*p* = 0.731), indicating that the remaining elevation is largely accounted for by TC levels. This underscores the need for caution when interpreting absolute CS concentrations in the context of differential lipid profiles across groups. Nevertheless, these findings should be interpreted with caution given the limited sample size of the Alzheimer’s disease subgroup. After age-matching, only 25 patients were available for analysis, substantially reducing statistical power and increasing the risk of both false-positive and false-negative findings. The residual disease effect, while statistically significant, may be unstable and requires independent validation in larger Alzheimer’s disease cohorts with more comprehensive covariate adjustment. Beyond its biomarker potential, elevated CS may exert detrimental effects through three interrelated mechanisms. First, CS binds to Aβ peptides with higher affinity than cholesterol and accumulates in plaques at levels approximately 50-fold higher than those in cell membranes, thereby accelerating plaque formation by up to 10-fold [8,9]. Second, CS rather than cholesterol specifically activates protein kinase C (PKC) α and δ isoforms, promoting tau hyperphosphorylation and fibrillar aggregation [10,11,13]. Third, SULT2B1b, the enzyme catalyzing CS synthesis, can sulphate endogenous LXR ligands to generate potent antagonists, thereby inhibiting LXR-mediated clearance of Aβ plaques, whereas LXR activation alone can reduce plaque burden by about 80% [29,30].

The lower CS levels observed in Crohn’s disease patients are intriguing. Previous studies have shown that SULT2B1 is compensatorily upregulated in the intestinal mucosa of patients with ulcerative colitis and that CS promotes colonic epithelial repair via the CS–NPC2–SREBP2–cholesterol synthesis axis [14]; in active inflammatory bowel disease (IBD), CS may therefore be excessively consumed for mucosal repair, leading to a decline in serum levels. While this hypothesis offers a plausible biological explanation, our findings indicate that the CS reduction in Crohn’s disease is largely driven by age confounding, consistent with the younger age profile of Crohn’s disease patients (median 28.0 years) and the known slight age-related increase in CS. Nevertheless, this interpretation is constrained by the limited sample size of Crohn’s disease subgroup. After age-matching (25–45 years), only 21 Crohn’s disease patients and 21 matched controls were available for analysis, rendering the statistical comparisons particularly underpowered. The *p*-value for the CS/TC ratio comparison is essentially non-significant, and the conclusion that the CS reduction in Crohn’s disease is predominantly driven by age confounding should not be overinterpreted. Conversely, given the small sample size, we cannot exclude the possibility that a genuine disease-specific effect exists but was not detected due to insufficient statistical power. The possibility of consumption-related CS decrease during active IBD, superimposed on age effects, warrants further validation in larger cohorts with stratification by disease activity.

At the glycemic level, the independent positive correlation between CS and HbA1c carries significant biological implications. HNF4α regulates SULT2B1b expression, forming a negative-feedback control loop in hepatic gluconeogenesis. As a negative-feedback signal of gluconeogenesis, elevated CS levels may reflect a compensatory response to hyperglycemic states [15]. Although CS concentrations in diabetic patients tended to be higher, the difference did not reach statistical significance, possibly owing to limited sample size and the dynamic changes in CS across different glycemic phases (compensated vs. decompensated). The independent positive correlation with glycated albumin further strengthens the link between CS and glucose metabolism. Notably, the age-stratified diagnostic analysis revealed a marked age dependency for T2M, suggesting that the value of CS as a glycemic marker may be more prominent in early-onset diabetes.

The most salient finding of this study is the independent, positive correlation between serum CS and both lipid parameters (TC, LDL-C, non-HDL-C, TG) and glycemic markers (HbA1c, GA-L), with partial correlation coefficients highest for TC and HbA1c. This provides crucial evidence for the metabolic pathway affiliation of CS. At the cholesterol metabolism level, CS, as a direct sulfated derivative of cholesterol, shares a common precursor pool [1,8]. Two seemingly contradictory views have emerged regarding CS’s regulation of cholesterol homeostasis: in ulcerative colitis, CS binds NPC2 to activate SREBP-2, upregulating cholesterol synthesis genes (e.g., *HMGCR*, *HMGCS1*, *CYP51*) and promoting de novo cholesterol synthesis in colonic epithelial cells to support mucosal repair [14]; conversely, recent work indicates that CS can lower intracellular cholesterol by promoting HMGCR degradation, reducing LDL-C uptake, and interfering with SREBP2 processing [31]. Our observation that CS correlates independently and positively with non-HDL-C is particularly noteworthy. Since non-HDL-C is considered a more comprehensive cardiovascular risk indicator than LDL-C [32,33], this positive association suggests that serum CS rises in parallel with atherogenic lipoprotein levels, possibly reflecting a compensatory increase in CS synthesis as a negative-feedback response to active cholesterol metabolism. Additionally, CS acts as a ligand for RORα, which in turn induces AMPK phosphorylation, suppresses LXRα activity, and reduces lipid accumulation in hepatocytes [34], further supporting CS’s involvement in systemic cholesterol homeostasis.

A broader methodological consideration arising from our findings is the inherent dependence of absolute CS concentrations on TC. Because CS constitutes only a minute fraction of the total cholesterol pool, group differences in CS may merely reflect differences in TC rather than disease-specific CS dysregulation. This is particularly relevant when comparing diseases with divergent age and lipid profiles—such as the younger, lower-TC Crohn’s disease group versus older controls. Our CS/TC ratio analyses support this concern: the Alzheimer’s disease elevation was no longer significant after TC normalization, although the Crohn’s disease analysis was limited by incomplete data. We therefore suggest that future studies should routinely report both absolute CS and CS/TC ratios and that statistical adjustments for TC—or normalization by TC—should be considered as a standard practice when evaluating CS as a biomarker across populations with differing metabolic baselines.

Despite these metabolic associations, the diagnostic utility of CS proved limited. After adjusting for TC and sex, CS provided no incremental diagnostic value beyond the baseline model, and its unadjusted discriminative ability fell below clinically acceptable thresholds for all diseases examined. These findings indicate that CS is not suitable as an independent diagnostic biomarker for any of the diseases evaluated; its potential role, if any, is restricted to a supplementary indicator of lipid metabolism, requiring cautious interpretation in the context of age and sex.

## 5. Limitations

Several limitations of this study should be acknowledged. First, the cross-sectional design precludes causal inference between CS and the diseases under investigation. Second, although healthy controls were defined based on physical examinations and routine laboratory data (including fasting blood glucose, renal function, and liver enzymes), we cannot completely rule out the presence of asymptomatic conditions—such as compensated subclinical endocrinopathies—that were not systematically screened (e.g., thyroid function tests), which may have introduced residual selection bias. Third, important covariates including body mass index (BMI), medication history (e.g., statin use), and disease severity scores (e.g., MMSE for Alzheimer’s disease, CDAI for Crohn’s disease) were not collected, potentially contributing to residual confounding. Of particular concern is the lack of detailed records on lipid-lowering agents, as such medications may directly influence both lipid profiles and CS levels, precluding us from fully disentangling intrinsic CS-lipid association from drug-induced alterations. Fourth, the sample size for each disease group was relatively small (39–71 cases), limiting statistical power for subgroup and interaction analyses. This is especially critical in the sex-stratified (approximately 20 cases per sex) and age-matched subgroup analyses, where estimates become highly sensitive to individual observations. Consequently, null findings in certain subgroups should not be equated with the absence of true effects, and significant findings—particularly in the Alzheimer’s disease subgroup—should be viewed as preliminary. Fifth, as a retrospective study influenced by real-world clinical practice, isolated disease presentations are relatively uncommon; thus, the possibility of mild or well-controlled comorbidities in these populations cannot be excluded. Sixth, although sex-specific reference intervals were established, age-specific intervals could not be reliably derived due to insufficient sample sizes in individual age groups (*n* ≈ 70–80 per group), falling short of the CLSI-recommended minimum of 120 subjects per partition for nonparametric methods. The modest age effect and its male-predominant nature further suggest that age-adjusted interpretation—rather than formal age-stratified intervals—is currently more appropriate. Finally, as a single-center study without external validation, the generalizability of our findings warrants caution. These limitations collectively underscore the need for validation in larger, multicenter cohorts with comprehensive covariate information and standardized reference methods.

## 6. Conclusions

In summary, this study establishes, for the first time, serum cholesterol sulfate reference intervals for a healthy Chinese population, with an overall range of 0.55–2.24 mg/L and clear evidence supporting sex-specific partitioning (males: 0.60–2.25 mg/L; females: 0.54–2.20 mg/L). CS levels are independently regulated by TC and sex, with a significant age–sex interaction whereby the age-related increase in CS is confined to males. Importantly, the apparent associations between CS and disease status were largely mediated by confounding from lipid profiles and age; after adjustment for these factors, CS possessed no independent diagnostic value. Therefore, CS is not suitable as a standalone diagnostic biomarker, but may serve as a supplementary indicator for assessing lipid metabolism, particularly in males where it may indirectly reflect age-related changes in cholesterol metabolism.

## Figures and Tables

**Table 1:** Linearity range of CS was evaluated across five independent analytical batches.

NC (mg/L)	Calculated Concentration (mg/L)					Mean	SD	Acc%	CV%
	B1	B2	B3	B4	B5				
10.00	9.73	9.81	9.77	9.79	9.89	9.80	0.06	97.97	0.63
5.00	5.18	5.08	5.11	5.09	5.04	5.10	0.05	101.96	1.01
2.00	2.17	2.18	2.18	2.18	2.15	2.17	0.02	108.56	0.72
1.00	0.91	0.90	0.93	0.95	0.92	0.92	0.02	91.94	2.14
0.50	0.51	0.51	0.52	0.51	0.51	0.51	0.01	101.88	0.96
0.20	0.23	0.22	0.21	0.21	0.21	0.21	0.01	106.60	4.92
0.10	0.09	0.11	0.09	0.09	0.09	0.09	0.01	92.00	11.19
0.05	0.05	0.04	0.05	0.05	0.05	0.05	0.01	98.80	12.34
Linear regression parameter									
Slope	1.06	1.05	1.053	1.05	1.07	1.055	0.10		0.90
R^2^	0.998	0.998	0.998	0.998	0.999	0.998	0.001		0.05

NC, nominal concentration; B, batch; SD, standard deviation; Acc%, accuracy%; CV%, coefficient of variation%.

**Table 2:** Extraction efficiency of CS.

Quality Control Level (mg/L)	Mean%	SD	CV%
2	111.17	0.01	1.09
0.5	98.48	0.01	1.33
0.1	107.80	0.08	7.80

SD, standard deviation; CV%, coefficient of variation %.

**Table 3:** Matrix effect.

Serum	Matrix	Mean Ratio	CV%	Bias%
1	Serum	10.73	0.01	
	Methanol	9.79	0.01	
	Mix	10.25	0.01	−0.08
2	Serum	13.68	0.01	
	Methanol	11.16	0.01	
	Mix	12.19	0.01	−1.88
3	Serum	12.87	0.01	
	Methanol	10.98	0.02	
	Mix	11.98	0.01	0.44

Mean ratio, the peak area ratio of CS to its internal standard; CV, coefficient of variation %; Mix, serum:methanol = 1:1. Bias = [*R*_mix_ − (*R*_serum_ +*R*_methanol_)/2]/[(*R*_serum_ +*R*_methanol_)/2], *R*_mix_ denotes the peak area ratio obtained from the mixed matrix (serum:methanol = 1:1, spiked post-extraction), and *R*_serum_ and *R*_methanol_ represent the corresponding ratios from pure serum matrix and pure methanol matrix, respectively.

## Data Availability

The original contributions presented in this study are included in the article. Further inquiries can be directed to the corresponding author.

## Supplementary Materials

The following supporting information can be downloaded at https://www.mdpi.com/article/10.3390/diagnostics16182953/s1, Table S1: Baseline comparisons of demographic characteristics and clinical laboratory parameters across all study groups; Table S2: Interaction effect between age and gender on CS levels; Table S3: Sex-specific correlations between age and serum CS; Table S4: Reference interval summary (non-parametric, 2.5th–97.5th percentile); Table S5: Spearman correlation and partial correlation analyses between CS concentration and clinically significant laboratory parameters; Table S6: Comparison of CS concentration levels between various disease groups and the healthy control group; Table S7: Results of the generalized linear model analysis for serum CS influencing factors (Gamma distribution, log-link function); Table S8: Summary of differences in univariate and multivariate analysis results; Table S9: Diagnostic performance of CS for various diseases after adjustment for TC and sex.
