# Supplementary material for: Establishment of Serum Cholesterol Sulfate Reference Intervals and Evaluation of Its Diagnostic and Metabolic Associations in Chinese Adults
Source: Diagnostics (Basel). 2026 Sep 12;16(18):2953. doi: 10.3390/diagnostics16182953 (PMC13605642; doi:10.3390/diagnostics16182953)
Supplement: Supplementary file 1 [file diagnostics-16-02953-s001.zip › diagnostics-4463387-supplementary.pdf]

Table S1: Baseline comparisons of demographic characteristics and clinical laboratory parameters across all study groups

Variable	HC (n=372)	OP (n=71)	AD (n=40)	OB (n=40)	DM (n=40)	NAFLD (n=40)	CD (n=39)	Overall p†
Age (years)	41.0 (31.0–51.0)	60.0 (50.0–68.0)	68.0 (61.0–72.2)	32.0 (25.8–38.2)	57.5 (46.2–76.0)	48.0 (36.0–58.8)	28.0 (23.0–37.5)	<0.001
Sex (M/F, %M)	181/191 (49%)	31/40 (44%)	20/20 (50%)	20/20 (50%)	20/20 (50%)	20/20 (50%)	19/20 (49%)	0.992
CS (mg/L)	1.29 (1.02–1.59)	1.35 (1.08–1.63)	1.49 (1.18–1.82)	1.23 (0.94–1.80)	1.28 (0.86–1.89)	1.25 (1.12–1.39)	1.13 (0.85–1.47)	0.026
TC (mmol/L)	4.35 (3.92–4.71)	4.60 (4.33–4.97)	5.07 (4.55–5.34)	4.89 (3.98–5.71)	4.14 (3.44–5.49)	4.57 (4.10–4.96)	3.55 (3.26–4.46)	<0.001
CS/TC	0.30 (0.24–0.37)	0.27 (0.24–0.34)	0.29 (0.24–0.34)	0.28 (0.23–0.31)	0.29 (0.24–0.38)	0.29 (0.23–0.32)	0.26 (0.24–0.33)	0.471
TG (mmol/L)	0.82 (0.63–1.08)	1.41 (1.00–2.13)	1.10 (0.86–1.69)	1.60 (0.99–1.97)	1.63 (1.02–2.22)	1.39 (0.90–2.00)	0.82 (0.73–1.08)	<0.001
HDL-C (mmol/L)	1.50 (1.30–1.76)	1.53 (1.15–1.77)	1.63 (1.33–1.94)	1.27 (1.10–1.52)	1.29 (1.06–1.50)	1.34 (1.02–1.53)	1.35 (1.08–1.78)	<0.001
LDL-C (mmol/L)	2.49 (2.14–2.83)	2.71 (2.38–2.89)	2.97 (2.32–3.25)	2.99 (2.15–3.63)	2.23 (1.69–3.29)	2.54 (2.26–3.02)	2.12 (1.82–2.72)	<0.001
non-HDL-C (mmol/L)	2.77 (2.41–3.16)	3.17 (2.71–3.58)	3.44 (2.74–3.88)	3.46 (2.64–4.24)	2.96 (2.25–3.88)	3.17 (2.71–3.79)	2.44 (2.00–2.95)	<0.001
GLU (mmol/L)	4.64 (4.42–4.93)	5.05 (4.90–5.47)	5.53 (5.00–5.86)	5.42 (5.04–6.07)	7.34 (6.64–8.21)	5.50 (5.28–6.06)	4.47 (4.26–4.67)	<0.001
HbA1c (%)	5.65 ± 0.30	5.88 ± 0.55	6.24 ± 0.86	5.96 ± 1.03	7.46 ± 1.54	6.02 ± 0.56	5.67 ± 0.28	<0.001
ALT (U/L)	14.00 (11.00–19.00)	19.00 (11.00–27.00)	15.00 (12.00–24.50)	25.00 (17.50–36.50)	21.50 (16.00–34.00)	29.00 (22.50–56.00)	12.00 (9.00–17.50)	<0.001
AST (U/L)	18.00 (16.00–21.00)	23.00 (19.00–26.00)	21.00 (17.50–25.50)	21.00 (17.00–26.50)	23.50 (19.00–29.25)	26.00 (21.50–34.50)	19.00 (14.50–22.00)	<0.001
GGT (U/L)	13.00 (10.00–18.00)	22.00 (14.75–29.00)	17.00 (13.50–22.00)	27.00 (18.50–52.00)	24.00 (14.75–48.75)	30.00 (20.00–46.50)	16.00 (12.00–24.50)	<0.001
TBIL (μmol/L)	10.70 (8.70–13.40)	10.70 (8.70–13.22)	12.60 (9.85–15.05)	11.60 (8.95–15.70)	12.25 (8.57–14.27)	13.90 (10.60–16.65)	11.10 (7.60–15.60)	0.021
ALB (g/L)	45.94 ± 1.93	45.61 ± 3.16	44.28 ± 2.86	47.51 ± 2.58	45.78 ± 5.32	47.42 ± 2.58	44.11 ± 5.50	<0.001
CREA (μmol/L)	63.00 (56.00–79.00)	73.00 (64.00–81.00)	73.00 (67.00–85.00)	72.00 (60.00–87.50)	85.50 (72.25–101.75)	66.00 (57.00–82.00)	67.00 (55.50–82.50)	<0.001
eGFR (mL/min/1.73m²)	108.25 (98.34–116.38)	91.90 (76.30–99.30)	83.70 (73.80–89.80)	109.35 (94.22–119.15)	81.80 (60.70–98.00)	105.40 (92.20–111.80)	119.70 (108.45–128.10)	<0.001
UA (μmol/L)	279.00 (237.00–324.00)	288.00 (260.00–357.00)	282.00 (236.50–324.00)	354.50 (297.50–420.75)	313.50 (269.50–358.00)	331.00 (258.00–385.00)	284.00 (239.50–323.50)	<0.001
UREA (mmol/L)	4.70 (4.10–5.60)	5.70 (4.60–6.70)	6.10 (5.10–6.70)	4.85 (4.20–5.62)	6.20 (5.33–7.80)	5.60 (4.90–6.45)	4.60 (3.50–5.50)	<0.001

Continuous variables are expressed as mean ± standard deviation (mean ± SD) or medians (IQR); categorical variables are expressed as frequencies (percentage).

† One-way ANOVA or Kruskal–Wallis test (for continuous variables) or χ^2 test (for gender); HC: healthy control; OP: osteoporosis; AD: Alzheimer's disease; OB: obesity; DM: diabetes mellitus; NAFLD: non-alcoholic fatty liver disease; CD: Crohn's disease.

Table S2. Interaction effect between age and gender on CS levels

Method	Main effect p (sex)	Main effect p (age)	Interaction p (sex and age)	Interaction F/β	Conclusion
Two-way ANOVA	0.036	<0.001	0.005	F=3.77	✓
GLM (Gamma log, continuous age)	0.038	0.625	0.002	β=0.102 (exp=1.107)	✓

✓ The interaction term is significant, indicating a synergistic effect between age and sex.

Table S3. Sex-specific correlations between age and serum CS

Gender	n	Age-bracket	Spearman ρ	Spearman p	Pearson r	Pearson p
Male	181	25-79	0.331	<0.001	0.3	<0.001
Female	191	22-82	-0.001	0.988	-0.031	0.672

Table S4. Reference Interval summary (non-parametric, 2.5th-97.5th percentile)

Group	n	Median (IQR)	P2.5(mg/L)	P97.5(mg/L)	Lower 90% CI	Upper 90% CI	Layered recommendations
All	372	1.29 (1.02–1.59)	0.55	2.24	0.5	2.35	Need for gender stratification
Male	181	1.35 (1.06–1.64)	0.60	2.25	0.54	2.42	Recommend
Female	191	1.23 (0.97–1.51)	0.54	2.20	0.48	2.32	Recommend

CI refers to the 90% confidence interval calculated using the Bootstrap method (2,000 bootstrap samples). Gender-based differences were assessed using the Mann–Whitney U test (p = 0.010).

Table S5. Spearman correlation and partial correlation analyses between CS concentration and clinically significant laboratory parameters

Indicator	Category	n	Spearman ρ	p	Partial correlation ρ (after adjusting for age and sex)	p	Independent correlation
non-HDL-C	lipids	548	+0.28	<0.001	+0.28	<0.001	✓
TC	lipids	548	+0.27	<0.001	+0.29*	<0.001	✓
LDL-C	lipids	548	+0.26	<0.001	+0.27	<0.001	✓
TG	lipids	548	+0.11	0.008	+0.09	0.028	✓
HbA1c	Glucose	446	+0.16	<0.001	+0.05	0.285	✗ (Spurious correlation)
GA-L	Glucose	172	+0.22	0.005	+0.24	0.002	✓
DBIL	Liver	572	−0.10	0.020	−0.14	<0.001	✓ (Negative Direction)
ALT	Liver	572	+0.12	0.004	+0.09	0.026	✓
AST	Liver	572	+0.09	0.041	+0.05	0.225	✗ (Spurious correlation)
CREA	Renal	599	+0.13	0.002	+0.03	0.453	✗ (Spurious correlation)
Mg	Bone/Mineral	181	+0.16	0.034	+0.13	0.073	✗ (Spurious correlation)
HCT	Blood	156	+0.16	0.041	-0.02	0.792	✗ (Spurious correlation)

✓ independent association; ✗ false association (age confounding); partial correlation adjusted for age and gender. TC, total cholesterol; non-HDL-C, non-high density lipoprotein cholesterol; LDL-C, low density lipoprotein cholesterol; TG, triglycerides; HbA1c, glycated hemoglobin A1c; GA-L, glycated albumin; DBIL, direct bilirubin; ALT, alanine aminotransferase; CREA, creatinine; Mg, magnesium; HCT, hematocrit.

Table S6: Comparison of CS concentration levels between various disease groups and the healthy control group

Disease	n	CS median (IQR)	Multiple ratio	Cohen's d	MWU p
AD	40	1.494 (1.183-1.816)	1.16	0.495	0.012
CD	39	1.134 (0.845-1.474)	0.88	-0.384	0.019
OP	71	1.350 (1.077-1.626)	1.05	0.219	0.183
DM	40	1.283 (0.859-1.888)	1.00	0.268	0.791
OB	40	1.228 (0.942-1.801)	0.95	0.136	0.978
NAFLD	40	1.250 (1.122-1.391)	0.97	-0.062	0.866

Multiplicative ratio = median value in the disease group / median value in the HC group.

Table S7: Results of the generalized linear model analysis for serum CS influencing factors (Gamma distribution, log-link function)

Variables	Standardized β	SE	z	p Value	CS Multiplication Factor Ratio	95%CI	Direction	marked
Male vs Female	0.125	0.033	3.81	0.0001	1.133	1.063-1.208	CS↑	Y
NAFLD vs HC	-0.138	0.075	-1.85	0.064	0.871	0.752-1.008	CS↓	N
DM vs HC	-0.135	0.099	-1.37	0.171	0.873	0.719-1.060	CS↓	N
CD vs HC	-0.121	0.104	-1.17	0.242	0.886	0.723-1.085	CS↓	N
TC (every 1SD)	0.084	0.019	4.41	<0.0001	1.088	1.048-1.129	CS↑	Y
OB vs HC	-0.067	0.095	-0.71	0.48	0.935	0.777-1.126	CS↓	N
HbA1c (every 1SD)	0.042	0.024	1.78	0.075	1.043	0.996-1.093	CS↑	N
OP vs HC	-0.041	0.085	-0.48	0.635	0.96	0.813-1.135	CS↓	N
CREA (every 1SD)	-0.031	0.02	-1.57	0.118	0.97	0.933-1.008	CS↓	N
GLU (every 1SD)	0.028	0.026	1.1	0.272	1.029	0.978-1.082	CS↑	N
AD vs HC	-0.027	0.117	-0.23	0.819	0.974	0.774-1.224	CS↓	N
ALB (every 1SD)	0.026	0.019	1.38	0.167	1.026	0.989-1.064	CS↑	N
HDL-C (every 1SD)	-0.025	0.02	-1.21	0.228	0.976	0.938-1.015	CS↓	N
GGT (every 1SD)	-0.024	0.022	-1.11	0.265	0.976	0.936-1.018	CS↓	N
Age (every 1SD)	0.022	0.018	1.25	0.212	1.023	0.987-1.059	CS↑	N
ALT (every 1SD)	0.018	0.02	0.92	0.356	1.019	0.980-1.059	CS↑	N
TBIL (every 1SD)	-0.011	0.017	-0.67	0.503	0.989	0.957-1.022	CS↓	N
TG (every 1SD)	-0.002	0.024	-0.08	0.94	0.998	0.953-1.046	CS↓	N
UA (every 1SD)	-0.002	0.02	-0.08	0.94	0.999	0.960-1.039	CS↓	N

Continuous variables were standardized (z-transformation); the CS multiple ratio = exp(β); model pseudo-R^2 = 0.153, AIC = 473.9, dispersion = 0.098; all VIFs <3.
HC: healthy control; OP: osteoporosis; AD: Alzheimer's disease; OB: obesity; DM: diabetes mellitus; NAFLD: non-alcoholic fatty liver disease; CD: Crohn's disease.

Table 8: Summary of differences in univariate and multivariate analysis results

Variable	Category	n	Single-factor (Spearman ρ/p)	Partial correlation r (adjusted for age + sex)/p	GLM odds ratio/p	Conclusion
TC	lipids	548	+0.266 / <0.001	+0.289 / <0.001	1.088 / <0.0001	Real Independent Effect
Sex (M vs F)	Demography	372	13.4	Correcting Variable	1.133 / 0.0001	Real Independent Effect
Age	Demography	372	171	Correcting Variable	1.023 / 0.212	TC-mediated attenuation of the univariate effect
HbA1c	Glucose	446	+0.157 / <0.001	0.178947368	1.043 / 0.075	Age confounding, pseudo-correlation
CREA	Renal	599	64	0.068432671	0.970 / 0.118	Age confounding, pseudo-correlation
ALT	Liver	572	30.25	3.576923077	1.019 / 0.356	Significant in partial correlation, but attenuated in GLM
TG	lipids	548	14.25	3.357142857	0.998 / 0.940	Significant in partial correlation, but attenuated in GLM
DBIL	Liver	572	-4.85	-0.139 / <0.001	Not included	Enhanced after correction (inhibitory effect)
AD vs HC	Disease	412	d=+0.495 / 0.012	—	0.974 / 0.819	TC/age-mediated spurious association
CD vs HC	Disease	411	d=-0.384 / 0.019	—	0.886 / 0.242	TC/age-mediated spurious association
NAFLD vs HC	Disease	412	d=-0.062 / 0.866	—	0.871 / 0.064	critical effect
OP vs HC	Disease	443	d=+0.219 / 0.183	—	0.960 / 0.635	No significant correlation
DM vs HC	Disease	412	d=+0.268 / 0.791	—	0.873 / 0.171	No significant correlation
OB vs HC	Disease	412	d=+0.136 / 0.978	—	0.935 / 0.480	No significant correlation

The single-factor Spearman's ρ represents the unadjusted correlation coefficient for the overall population; the partial correlation coefficient r denotes the Spearman's ρ calculated after OLS residual adjustment (corrected for age and gender); GLM refers to a Gamma-family log-link model, where the multiplicative factor is given by exp(β); for continuous variables, the interpretation is "per 1 SD change"; for categorical variables, the comparison is made against the reference group. DBIL was excluded from the primary GLM model due to its collinearity with TBIL.

Table S9: Diagnostic performance of CS for various diseases after adjustment for TC and sex

Disease	AUC (CS alone)	Baseline AUC (TC + sex)	Full model AUC (+CS)	ΔAUC	DeLong p	AUC (CS residual)	LR p
AD	0.620	0.762	0.759	-0.004	1.000	0.574	0.333
CD	0.614	0.668	0.663	-0.005	0.914	0.634	0.077
OP	0.550	0.677	0.677	0.000	1.000	0.53	0.981
DM	0.513	0.505	0.535	0.031	0.702	0.552	0.089
OB	0.499	0.650	0.655	0.004	1.000	0.476	0.660
NAFLD	0.508	0.616	0.623	0.007	1.000	0.523	0.522

Baseline model = TC + sex (logistic regression); Full model = TC + sex + CS; ΔAUC = full model AUC – baseline AUC; DeLong's p-value = p-value comparing the AUCs of the baseline model and the full model; CS residual AUC = ROC AUC of the residual values after regressing CS against TC + sex; LR p-value = p-value from the likelihood ratio test. HC: healthy control; OP: osteoporosis; AD: Alzheimer's disease; OB: obesity; DM: diabetes mellitus; NAFLD: non-alcoholic fatty liver disease; CD: Crohn's disease.
